# Synonymous codon usage bias is correlative to intron number and shows disequilibrium among exons in plants

**DOI:** 10.1186/1471-2164-14-56

**Published:** 2013-01-28

**Authors:** Zhen Qin, Zhengqiu Cai, Guangmin Xia, Mengcheng Wang

**Affiliations:** 1 The Key Laboratory of Plant Cell Engineering and Germplasm Innovation, Ministry of Education, School of Life Science, Shandong University, 27 Shandanan Road, Jinan, Shandong, 250100, China; 2 The Center for Biomedical Informatics, Harvard Medical School, 10 Shattuck Street, Boston, Massachusetts, 02115, USA

**Keywords:** Synonymous codon usage bias, Plant evolution, Intron number, Exon position, DNA methylation

## Abstract

**Background:**

Evidence has been assembled to suggest synonymous codon usage bias (SCUB) has close relationship with intron. However, the relationship (if any) between SCUB and intron number as well as exon position is at present rather unclear.

**Results:**

To explore this relationship, the sequences of a set of genes containing between zero and nine introns was extracted from the published genome sequences of three algal species, one moss, one fern and six angiosperms (three monocotyledonous species and three dicotyledonous species). In the algal genomes, the frequency of synonymous codons of the form NNG/NNC (codons with G and C at the third position) was positively related to intron number, but that of NNA/NNT was inversely correlated; the opposite was the case in the land plant genomes. The frequency of NNC/NNG was higher and that of NNA/NNT lower in two terminal exons than in the interstitial exons in the land plant genes, but the rule showed to be opposite in the algal genes. SCUB patterns in the interstitial and two terminal exons mirror the different evolutionary relationships between these plant species, while the first exon shows the highest level of conservation is therefore concluded to be the one which experiences the heaviest selection pressure. The phenomenon of SCUB may also be related to DNA methylation induced conversion of CG to AT.

**Conclusions:**

These data provide some evidence of linkage between SCUB, the evolution of introns and DNA methylation, which brings about a new perspective for understanding how genomic variation is created during plant evolution.

## Background

The degeneracy of the nucleotide triplet code, is such that, with the exceptions of Met and Trp, each amino acid residue is encoded by two or more synonymous codons (SCs). SC frequency can vary from one genome to another, and even from one gene to another within a single genome [1]. The resulting variation has been termed “synonymous codon usage bias” (SCUB) and has been identified in prokaryotic organism genomes as well as in those of both animals and plants. The evolution of SCs is proposed to reflect a balance between mutation, genetic drift and natural selection [2,3].

Evidence has been assembled to suggest a relationship between intron and SCUB (see review by [1]). The gain/loss of introns is a key component of the evolution of genomes [4,5], via either transposon insertion [6] or “reverse splicing” [7], but also as a by-product of recombinational error [8]. Indel events necessarily entail prior DNA breakage and refusion, processes associated with genomic shock [9,10], a consequence of which can be the induction of local single nucleotide polymorphisms. Just as is the case for indels, the gain/loss of introns also potentially induces genomic shock and its attendant consequences [11]. The propensity for intron gain/loss is related both to intron number and the intron’s position within the gene [12], so there is reason to suspect that SCUB may in turn also be related to these variables.

The presumed ancestors of land-based plants, from mosses to angiosperms, are the single celled algae. Polyploidization has been one of the major drivers of genome evolution. The process of genome duplication can result in orthologous genes evolving a different intron content, and in so doing can contribute to the divergence in gene structure between species [5]. For example, DNA replication slippage and repetitive sequence duplication are thought to be the major sources of intron gain [5,13]; segmental genome duplication can generate a functional intron that could be deleted during RNA editing [14]. Thus, there may well be an association between SCUB and the patterns of plant evolution; but as to whether or not SCUB based on intron number and exon position could shed new light on the evolutionary path of plants has not yet been fully evaluated. Here we have based a study of SCUB on the genome sequences of three species of algae, one of moss, one of fern and six angiosperms (three monocotyledonous species, three dicotyledonous species). Our aim was to identify the correlation, if any, between SCUB and both intron number and exon position.

## Results

### Intron distribution and gene length

A comparison of the genomes of the three algal species (*Ectocarpus fasciculatus*, *Chlamydomonas reinhardtii*, *Volvox carteri*), the moss (*Physcomitrella patens*), the fern (*Selaginella moellendorffii*) and the six angiosperms (*Oryza sativa*, *Zea mays*, *Sorghum bicolor*, *Arabidopsis thaliana*, *Glycine max* and *Populus trichocarpa*) showed that, the number of genes is reduced as the frequency of introns increases (Additional file 1: Figure S1). The proportions of genes containing 0–9 introns ranged from 73.6% in *C. reinhardtii* to 90.1% in *P. trichocarpa*.

### The correlation between SCUB frequency and intron number

**Figure 1:**
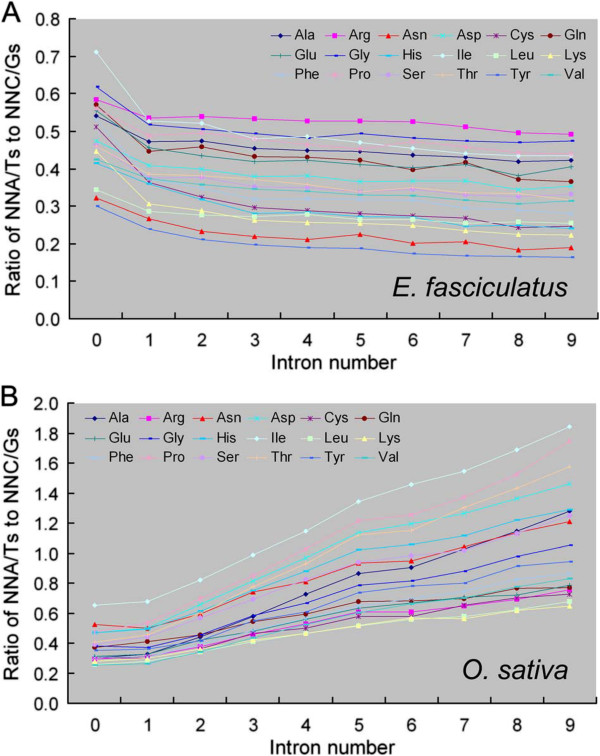
**The ratio between the frequencies of NNA/Ts to NNC/Gs is correlated differently with intron number between (A) *E. fasciculatus *and (B) *O. sativa.*** NNA/Ts: frequency of SCs having an A and a T in the third position, NNC/Gs: frequency of SCs having a C and a G in the third position. The equivalent ratios for the other genomes investigated are shown in Additional file 3: Figure S2.

The SCUB frequency in genes bearing 0–9 introns was based on the analysis of 59 codons. SCs formed by the alternate presence of an A or a T in the third position (NNAs, NNTs) behaved quite distinctly from NNCs and NNGs (Additional file 2: Table S1). In the algal genomes, the frequencies of most NNAs and NNTs were inversely related to intron number, while the relationship was largely opposite for most NNCs and NNGs. In the land plant sequences, however, the frequencies of NNAs and NNTs were positively correlated with intron number but the opposite was the case for NNCs and NNGs. These trends were depicted graphically with respect to the ratios of NNAs and NNTs to NNCs and NNGs of 18 SCs, which were positively related to intron number in land plants, but negatively in algae (Figure 1, Additional file 3: Figure S2).

**Figure 2:**
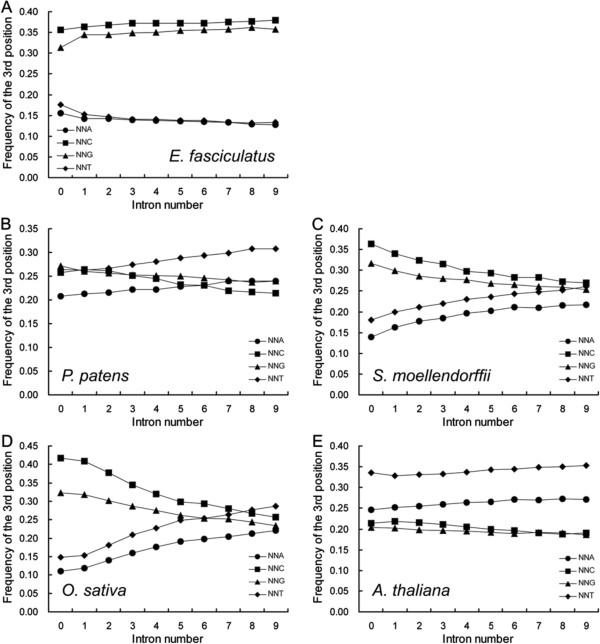
**SC frequency based on the third nucleotide is correlated differently with intron number in algal (A) and land plant (B-E) genomes.** NNA, NNT, NNC and NNG are defined as the ratio of total SCs with A, T, C and G at the third position, respectively to 59 SCs. The equivalent relationships in the other genomes investigated are shown in Additional file 4: Figure S3.

The algal and the land plant genomes also differed with respect to the global frequency of NNA, NNT, NNC and NNG, which were defined as the ratios of the frequencies of all NNAs, NNTs, NNCs and NNGs to 59 SCs, respectively. In the former genomes, both NNC and NNG were commoner than NNA and NNT, and their frequencies were correlated with intron number in the same way as were NNAs, NNTs and NNCs, NNGs, respectively (Figure 2A). In the land plant genomes, both the NNA and the NNT frequencies rose as intron number increased, while those of NNC and NNG both fell (Figure 2B-E). In the moss genome, the frequency of each of these four codon types was similar across the fully gene set (Figure 2B), while in *S. moellendorffii* and the three cereal genomes, the NNC and NNG frequencies were notably higher than those of NNA and NNT in genes carrying 0–4 introns, and similar in genes carrying 5–9 introns (Figure 2C,D, Additional file 4: Figure S3). Among the three dicotyledonous species genomes, the excess of NNA and NNT was more apparent than that of NNC and NNG in the whole gene set, but particularly when the number of introns was large (Figure 2E, Additional file 4: Figure S3).

### SCUB frequency is variable within exonic sequence

**Figure 3:**
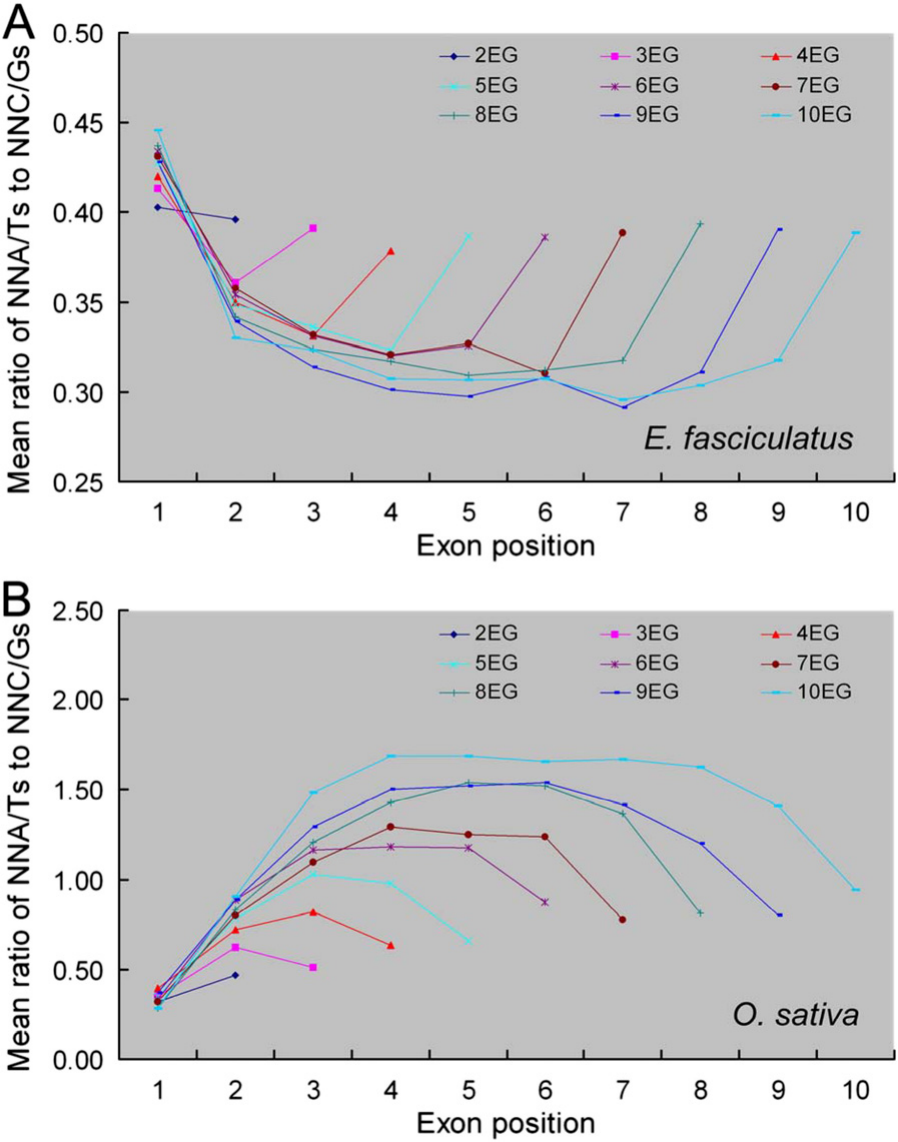
**The Mean of NNA/Ts to NNC/Gs ratios within SCs differs between (A) *E. fasciculatus *and (B) *O. sativa.*** NNA/Ts: frequency of SCs having an A and a T in the third position of an amino acid, NNC/Gs: frequency of SCs having a C and a G in the third position of an amino acid. The mean is defined as the average of NNA/Ts to NNC/Gs ratios of 18 amino acids with SCs. 2EG-10EG: gene sequences arranged into between two and ten exons. The equivalent ratios in the other genomes investigated are shown in Additional file 5: Figure S4.

**Figure 4:**
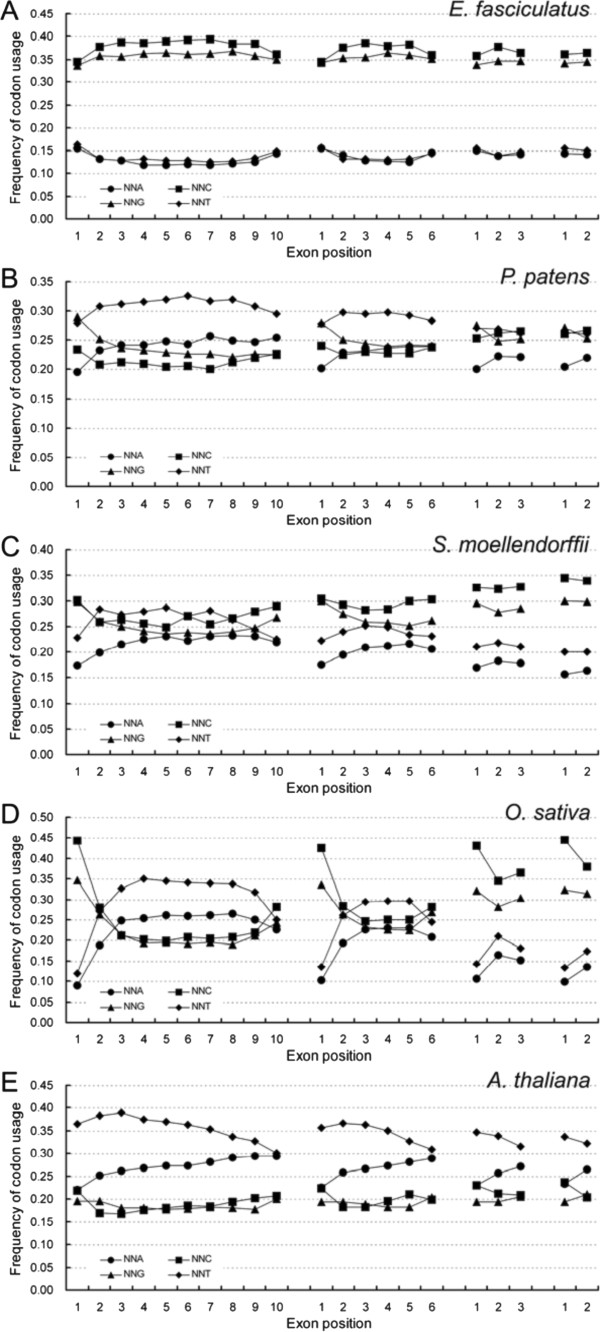
**SC frequency based on the third nucleotide shows disequilibrium among exons and differs between algal (A) and land plant (B-E) genomes.** NNA, NNT, NNC and NNG are defined as the ratio of total SCs with A, T, C and G at the third position, respectively to 59 SCs. The equivalent ratios in the other genomes investigated are shown in Figure S5.

In genes containing between two and ten exons, the SC frequencies showed arched-curves (‘∩’ or ‘∪’), interstitial exons had higher or lower frequencies than two terminal exons from the first to the last exons in both algae and land plants (data not shown). NNCs and NNGs mostly possessed ‘∪’ curves, but NNAs and NNTs mostly appeared ‘∩’ patterns in land plants; the pattern in the algal genomes was the opposite. In each of 18 amino acids with SCs, the ratio between the frequency of NNAs, NNTs to that of NNCs, NNGs among exons displayed a ‘∩’ distribution in land plants but a ‘∪’ distribution in algae (data not shown), and the mean value of such ratios among the 18 SCs was also characterized by the similar patterns in either the land plants or the algae (Figure 3, Additional file 5: Figure S4). Similarly, the individual frequencies of NNA, NNT and NNC, NNG appeared ‘∩’ and ‘∪’ distributions respectively across the whole set of exons in the land plants, and the opposite is shown in the algae (Figure 4, Additional file 6: Figure S5).

The mean ratios of NNAs, NNTs to NNCs, NNGs within the first exon were comparable among genes with 2–10 exons in either algal or land genomes; in comparison with the first exon, these ratios in the subsequent exons were higher in the land plant but lower in the algal genes (Figures 3, Additional file 5: Figure S4). In the final exon, the ratios were conserved among the algal genes, but were positively correlated with intron number among the land plant genes; this correlation was weakest among the angiosperm species. In the interstitial exons, the ratios were conserved among the algal genes, but were variable among the land plants, particularly in genes having a larger number of introns. Heterogeneity between exons was also reflected by the frequencies of NNA, NNT, NNC and NNG (Figure 4), which were relatively well conserved in the first exon across all the test species. Conservation was good in the final exon among the algal species; the frequency of NNC and NNG was positively correlated with intron number in the moss, fern and monocotyledonous angiosperm species, but that of NNA and NNT was negatively correlated; among the dicotyledonous species, the frequency of NNC and NNG was well conserved, but that of NNA was reduced and that of NNT was increased in genes carrying a larger number of introns.

### The role of DNA methylation in the formation of SCs

**Figure 5:**
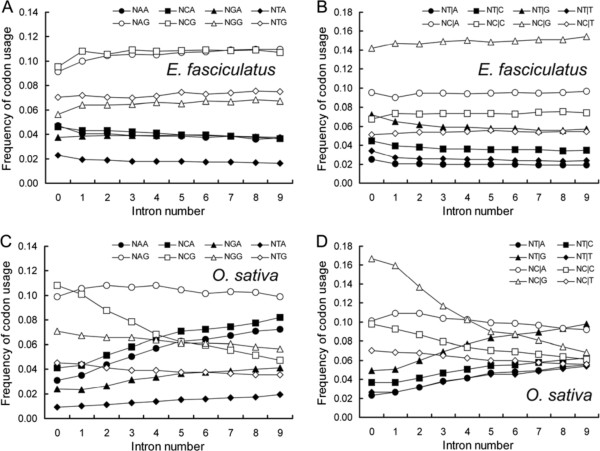
**The association between the DNA methylation induced conversion of C to T and SCUB based on intron number. A**, **C**: SC frequencies based on the second-third nucleotide combinations (N**NN**); **B**, **D**, SC frequencies based on the third-next codon’s first nucleotide combinations (N**N**|**N**). The equivalent associations in the other genomes investigated are shown in Additional file 7: Figure S6 and Additional file 8: Figure S7.

DNA methylation is a major source of DNA variation, since methylated C can readily be converted into T [15]. The conversion of methylated C in CpG or its complement strand produces TpG or CpA, and the conversion of two cytosines produces TpA. To investigate the influence of C methylation on the relationship between SCUB and either intron number or exon position, the frequencies of 16 second-third nucleotide combinations (N**NN**) and 16 third-next codon’s first nucleotide combinations (N**N**|**N**) were compared. In the land plant genomes, an increase in intron number was associated with a sharper fall in the frequency of NCG than in that of either NAG, NTG or NGG, while the frequency of NCA raised with stronger extent than the other N**NA** triplets (Figure 5C, Additional file 7: Figure S6); the frequencies of the four N**NC** and the four N**NT** codons did not differ from one another (data not shown). The decline in the frequency of NC|G was steeper than that of the other three possible N**C**|**N**s in those land plant genes with a high intron number; at the same time, the frequency of NT|G ascend more sharply than other N**T**|**N**s (Figures 5D, Additional file 8: Figure S7). This behaviour was not shown by either N**G**|**N** or N**A**|**N** (data not shown). Unlike NC|A and NT|G, the frequencies of NTA, NTG, NC|A and NT|A were largely conserved, presumably reflecting a strong level of selection pressure against base conversion at the first and second positions of the codon.

**Figure 6:**
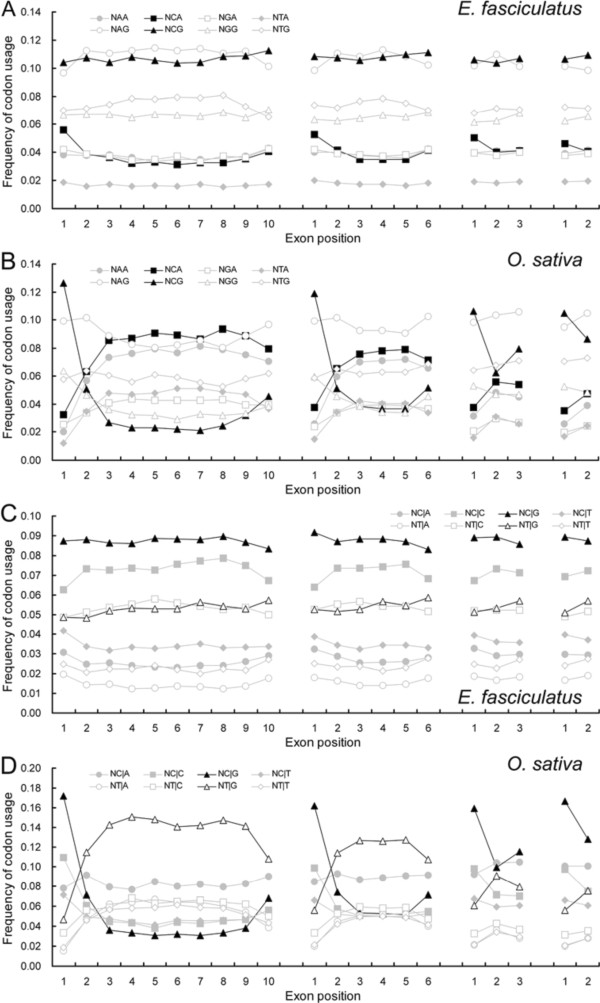
**The association between the DNA methylation induced conversion of C to T and SCUB based on exon position. A**, **C**: SC frequencies based on the second-third nucleotide combinations (N**NN**); **B**, **D**, SC frequencies based on the third-next codon’s first nucleotide combinations (N**N**|**N**). The equivalent associations in the other genomes investigated are shown in Additional file 9: Figure S8 and Additional file 10: Figure S9.

A relationship between DNA methylation-induced nucleotide substitution and SCUB was also detectable among the exon sequences in the land plant genomes (Figure 6, Additional file 9: Figure S8, Additional file 10: FigureS9). The distribution of NCG frequencies from the first to the last exons had larger ‘∪’ curvatures than those associated with the other N**NG**s - the frequencies of NAG, NGG and NTG were rather constant among the various exons. The behaviour of NCA was rather similar to that of NCG, and its distribution showing the largest ‘∩’ curvatures among the N**NA**s. The curvatures associated with NC|G and NT|G distribution appeared to be greater than those associated with either the N**C**|**N**s or the N**T**|**N**s (Figure 6C,D), while those associated with either the N**NC**s and N**NT**s or the N**A**|**N**s and N**G**|**N**s were similar to one another. In comparison with the other N**NG**s and N**C**|**N**s, the frequencies of NCG and NC|G were the most closely positively correlated with those of, respectively, NNG and NNC, and the most negatively with those of NNA and NNT. Similarly, compared to the other N**NA**s and N**T**|**N**s, the frequencies of NCA and NT|G were most strongly positively correlated with those of, respectively, NNA and NNT, and most strongly negatively with NNG and NNC (data not shown).

The role of methylation in SCUB is also revealed by frequencies of SCs within a certain amino acid (Additional file 2: Table S1). For the residues Ala, Pro, Ser and Thr, each of which is encoded by more than two SCs each with a C in its middle position, the NCG frequency declined more sharply than that of NCC as the intron number increased, while the NCA frequency rose more obviously. For Arg, Gly, Leu and Val (codons without a C in the middle position), the frequencies of NNCs were clearly lower than those of NNGs, while those of NNTs was higher than those of NNAs. A comparison between the pairs of residues Asn *vs* Lys, Asp *vs* Glu and Gln *vs* His (the first two nucleotides of the SCs lacking C at the second position are the same in each pair) showed that the frequencies of NNCs and NNTs had more distinguishable alteration than NNGs and NNAs, respectively. A similar analysis of asymmetric methylation, based on the codons CHG and CHH (H = A, C or T) was carried out by assessing the frequencies of **N**|**NN** and **NNN**, and a more obvious alternation in **C**|**NN** and **NNG** frequencies than in others was found based on both intron number and exon position (data not shown). Unlike for the land plant genomes, in the algal genomes the frequencies of NCG and NC|G, and of NCA and NT|G were not different from those of N**NN** and N**N**|**N**, and were uncorrelated with both intron number and exon position (Figure 5A,B, 6A,B, Additional file 8: Figure S7, Additional file 9: Figure S8, Additional file 10: Figure S9).

### Plants are clustered with respect to SCUB based on intron number and exon position

**Figure 7:**
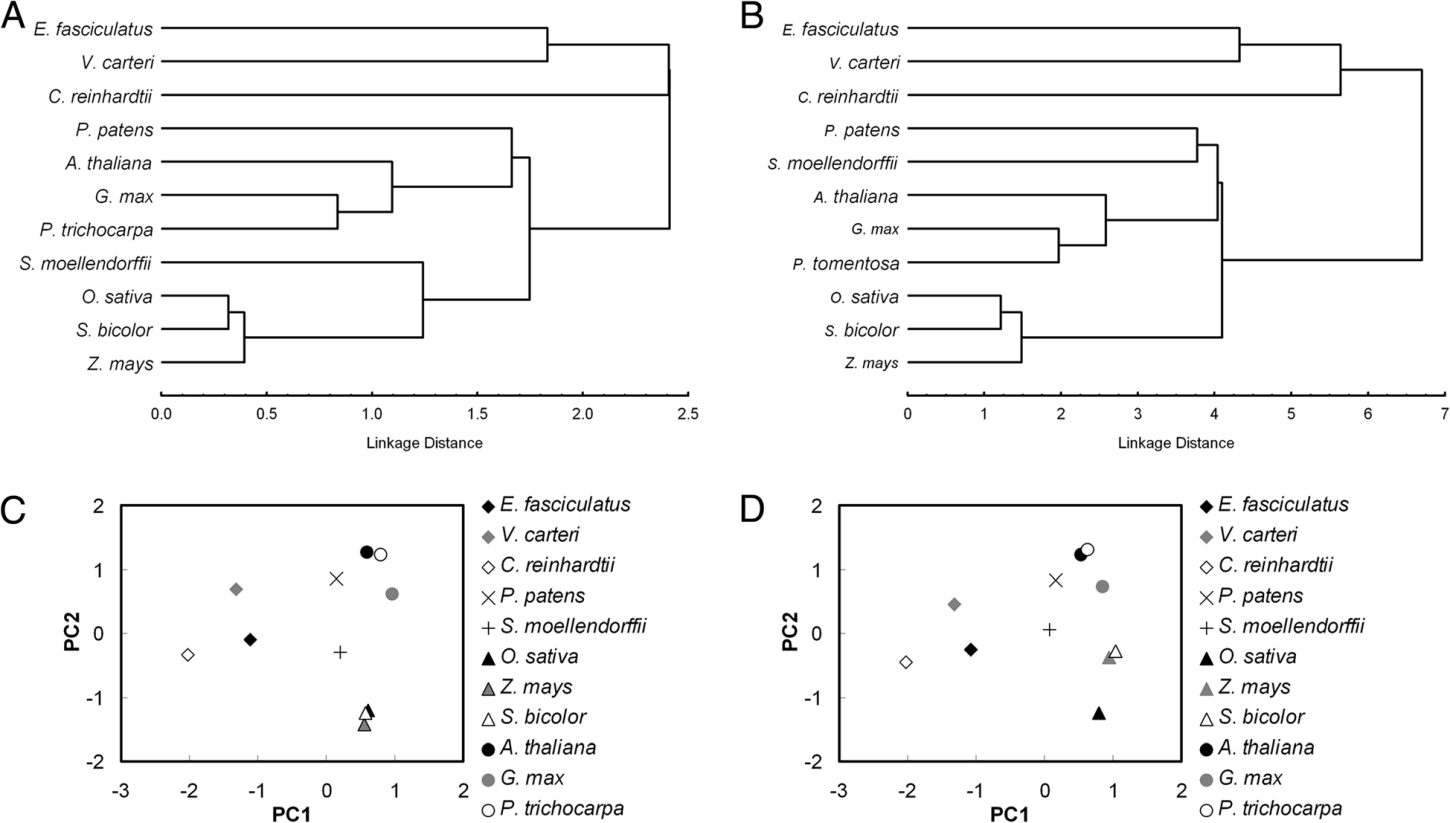
**Cluster and PC analysis of SCUB frequencies based on intron number and exon position in algal and land plant genomes.** (**A**, **B**) Cluster analysis, (**C**, **D**) Principal component analysis. PC1, PC2: coefficients associated with the first two extracted principal components. **A**, **C**: SC frequency in genes comprising from zero to nine introns. **B**, **D**: SC frequency in exons of gene sequences arranged into between two and ten exons.

SCUB frequency clearly distinguished the algae from the land plants (Figure 7). Within the latter group of species, a principal component (PC) analysis based on either intron number or exon position also divided the monocotyledonous and dicotyledonous species into two recognizable clades (Figure 7C,D), although the relationships between the land plant species was somewhat different when a clustering analysis was applied as an alternative to the PC (Figure 7A,B). A PC analysis using SCUB frequency at various exon positions suggested a level of heterogeneity to be present (Additional file 11: Figure S10). SCUB frequency based on the full set of exons successfully separated the algal from the land plant genomes; that based on the first exon only produced four groups (algae, mosses/ferns, monocotyledonous species and dicotyledonous species); that based on the last exon alone merged the two angiosperm families into a single group; finally, that based on the interstitial exons produced three clades, namely the algae, moss/fern/monocotyledonous species and the dicotyledonous species.

## Discussion

### Intron evolution is a strong driver of SCUB in land plants

Intron loss is a major feature of eukaryotic evolution [16,17]. Changes in the intron structure can induce mutations in adjacent exons, forming either SCs or non-synonymous codons that lead to a bias towards lower GC content [18]. The present analysis has suggested that genes with fewer introns tend to show a heightened frequency of NNC and NNG and a concomitant lowered frequency of NNA and NNT codons. Genes with fewer introns are thought to be favored by selection, and to evolve more slowly [19], with the result that the GC content of the exonic fraction of the genome has tended to have risen over time [20]. Thus it is possible that SCUB is directed to GC preference in genes with less introns that occur lower frequency of single-nucleotide substitution induced by intron evolution.

The single nucleotide changes induced by indel formation can occur over a distance of several hundred bases from the site of the indel itself [11], and the substitution level is negatively correlated with its distance to the indel [21]. Thus, the gain or loss of an intron is only likely to induce single nucleotide change in the flanking exons. Since intron gain/loss takes place preferentially at the 3^′^ terminus of eukaryotic genes [22,23], the implication is that GC enrichment at the 5^′^ terminus of exons is not likely to be greatly affected by intron evolution. The first exon does in fact tend to be the most enriched with respect to NNC and NNG, at least in land plant genes, and the frequency of these codons occurs is largely independent of how many exons are present. Thus it is the first exon which experiences the most intense selection pressure, and it is therefore this exon which remains most highly conserved. We have demonstrated that the frequency of NNC and NNG codons in the final exon appears to be higher than in the interstitial exons. If it is the case that indels tend to occur most readily in regions where the GC content is relatively low [21,24] and that their effect is to reduce GC content [18], then this represents a contradiction with the proposal that intron evolution (and especially intron loss) is most rapid at the 3^′^ terminus of genes [8]. The present analysis suggests rather that intron evolution is most rapid in the interstitial exons, consistent with the observation that a large proportion of intron loss is experienced in the middle of gene sequences [5].

### SCUB allows insights into plant evolution

The algae arose long before the appearance of land plants, and had already been exposed to a long period of selection which would have tended to favor GC enrichment [25]. Our analysis of three algal genomes has shown a marked SC bias of NNC and NNG over NNA and NNT (Figures 2, 4). A possible inference from this observation is that algal genomes have become very stable and that intron evolution now is very much slowed. Polyploidization has been a ubiquitous process in the evolution of higher plants. It induces a range of genomic shock associated events, such as gene loss and single nucleotide changes [26]. The latter are heavily biased towards A and T [27]. A salient property of enlarged genomes is that they provide buffering against selection pressure [17,28], and such a reduction favors the enrichment of the genome’s GC content. These two processes together could account for the observed shift in SCUB from NNC and NNG to NNA and NNT in land plant genes, a shift which is most pronounced in the dicotyledonous species (Figure 2).

Both the divergence of the gymnosperms and angiosperms from the ferns, and that of the angiosperms from the gymnosperms involved whole genome duplication events [26]. The dependence of SCUB pattern on intron number is comparable between the fern and the monocotyledonous species (Figure 2), so does not reflect a major effect of either of these genome-wide events. The marked preference for NNA and NNT among the dicotyledonous species is suggestive of the influence of polyploidization events occurring post the divergence of the monocotyledonous and dicotyledonous species [29-36]. SCUB based on exon position mirrors very closely the important events which have driven plant evolution (Figure 7). The cluster pattern of algal and land plant species based on either the first, last or interstitial exons (Additional file 11: Figure S10) both resembles the presumed chronology of plant evolution, and suggests a degree of SCUB heterogeneity.

### DNA methylation contributes to SCUB during intron evolution

The formation of indels contributes to the level of DNA methylation [11]. The DNA methylation induced conversion of CG to AT is thought to be a potent agent of naturally occurring mutagenesis [37]. The present data has shown that changes in the frequencies of both NNC and NNG dependent on either intron number or exon position are well correlated with those of, respectively, NCG and NC|G (Figure 5,6). The implication is that the increased rate of intron evolution associated with genes having a higher number of introns drives up the likelihood of DNA methylation and therefore generates a bias towards NNA and NNT. This bias is more recognizable in the interstitial exons than in the two outermost ones, so implies that intron evolution is favored in the interstitial region of genes. DNA methylation thus is likely to be a major driver of SCUB during intron evolution.

## Conclusions

SCUB is correlated with intron number and is non-homogeneous across all exons. The pattern of its heterogeneity differs from plant species to plant species. It has also been shown that DNA methylation is likely a major driver of SCUB. These inferences provide a new perspective for understanding how genomic variation is created during plant evolution. As yet it is unclear whether or not animal genomes behave in the same way as plant genomes appear to.

## Methods

### Genome sequences

The genome sequences of *O. sativa* and *A. thaliana* were downloaded from http://www.ncbi.nlm.nih.gov/genome, that of *S. moellendorffii* from http://genome.jgi-psf.org/Selmo1/Selmo1.download.ftp.html, *E. fasciculatus* from https://bioinformatics.psb.ugent.be/webtools/bogas/. and other species from http://www.plantgdb.org/XGDB/phplib/download.php.

### Gene structure

The intron/exon structure of the *O. sativa* and *A. thaliana* genes was obtained from the CDS annotation, while *E. fasciculatus* genes were identified from their cds file and their structure was inferred from the relevant gff3 file. For the other species, gene sequences were extracted from the appropriate nucleotide fa files, and their structure from the relevant gff3 files. For genes which encoded more than one transcript, the intron structure was inferred from the sequence of the primary transcript. ATG triplets were taken as start codons, and TAA, TGA and TAG as stop codons [38]. The codon separated by a intron between the first and the second nucleotides was acted as the condon of the intron’s 3^′^-adjacent exon, while that separated between the second and the third nucleotides belonged to the 5^′^-adjacent exon. These analyses were performed using a customized Pearl script.

### Calculation of SCUB frequency

Calculations were based on 59 (of the possible 64) codons, encoding 18 amino acids; the five not considered comprised the three stop codons, ATG (Met) and TGG (Trp). The SC frequency of a given residue was defined as the ratio between the number of a given SC to the number of all SCs for that particular amino acid. The SC frequency based on the third position nucleotide (NNA, NNT, NNC and NNG) was given by the ratio of the number of SCs having a given nucleotide to the total number of 59 codons. The SC frequency of the second/third nucleotide combinations (N**NN**) and the third nucleotide/first nucleotide of the following codon (N**N**|**N**) was defined as the number of a certain combination to the total number of 59 codons.

### Cluster and PC analysis

SC frequencies were subjected to both a cluster analysis based on the joining tree method implemented within the STATISTICA software package (V6.0, StatSoft) and a PC analysis based on the varimax method implemented within the SAS software package (V8.0, SAS Institute Inc.). Scatter plot diagrams were generated from the coefficients given by the first two PCs.

## Abbreviations

SC: Synonymous codon; SCUB: Synonymous codon usage bias; NNA, NNC, NNG, NNT: Synonymous codons with A, C, G and T at the third position; N**NN**: the synonymous codon combinations based on the second-third nucleotides; N**N**|**N**: the synonymous codon combinations based on the third nucleotide of the codon and the first nucleotide of the next codon.

## Competing interests

The authors declare that they have no competing interests.

## Authors’ contributions

ZQ developed the programs and analyzed the data, ZC developed the programs, GX designed the experiment and improved the paper, MW analyzed the data and wrote the paper. All authors read and approved the final manuscript.

## Supplementary Material

Additional file 1: Figure S1 The frequency of introns in the 11 plant genomes investigated. Click here for file

Additional file 2: Table S1 The correlation between SCUB frequency and intron number. Click here for file

Additional file 3: Figure S2 The relationship between NNA/Ts : NNC/Gs ratio and intron number in the 11 test species. NNA/Ts: frequency of SCs having an A and a T in the third position, NNC/Gs: frequency of SCs having a C and a G in the third position. Click here for file

Additional file 4: Figure S3 The relationship between the SC frequency based on the third position and intron number in the 11 test species. NNA, NNT, NNC and NNG are defined as the ratio of total SCs with A, T, C and G at the third position, respectively to 59 SCs. Click here for file

Additional file 5: Figure S4 The relationship between NNA/T : NNC/G ratio and exon position in the 11 test species. NNA/Ts: frequency of SCs having an A and a T in the third position of an amino acid, NNC/Gs: frequency of SCs having a C and a G in the third position of an amino acid. The mean is defined as the average of NNA/Ts to NNC/Gs ratios of 18 amino acids with SCs. 2EG, 3EG … indicates the number of exons present in the gene sequence (2, 3 …). Click here for file

Additional file 6: Figure S5 The relationship between the frequency of codon usage (NNA, NNC, NNG and NNT) and exon position in the 11 test species. NNA, NNT, NNC and NNG are defined as the ratio of total SCs with A, T, C and G at the third position, respectively to 59 SCs. Click here for file

Additional file 7: Figure S6 The relationship between the frequency of codon usage within a codon (NAA, NCA, NGA, NTA, NAG, NCG, NGG and NGT) and intron number in the 11 test species. SC frequencies based on the second-third nucleotide combinations were calculated. Click here for file

Additional file 8: Figure S7 The relationship between the frequency of codon usage between adjacent codons (NT|A, NT|C, NT|G, NT|T, NC|A, NC|C, NC|G and NC|T) and intron number in the 11 test species. SC frequencies based on the third-next codon’s first nucleotide combinations were calculated. Click here for file

Additional file 9: Figure S8 The relationship between the frequency of codon usage (NAA, NCA, NGA, NTA, NAG, NCG, NGG AND NTG) and exon position in the 11 test species. SC frequencies based on the second-third nucleotide combinations were calculated. Click here for file

Additional file 10: Figure S9 The relationship between the frequency of codon usage (NC|A, NC|C, NC|G, NC|T, NT|A, NT|C, NT|G and NT|T) and exon position in the 11 test species. SC frequencies based on the third-next codon’s first nucleotide combinations were calculated. Click here for file

Additional file 11: Figure S10 Principal component analysis of SCUB frequency based on exons at various positions in the 11 test species. (A) All exons, (B) first exon only, (C) interstitial exons, (D) last exon only. PC1, PC2: coefficients associated with the first two extracted principal components. Click here for file
