# Supplementary figures and images for: Synonymous codon usage bias is correlative to intron number and shows disequilibrium among exons in plants
Source: BMC Genomics. 2013 Jan 28;14:56. doi: 10.1186/1471-2164-14-56 (PMC3576282; doi:10.1186/1471-2164-14-56)

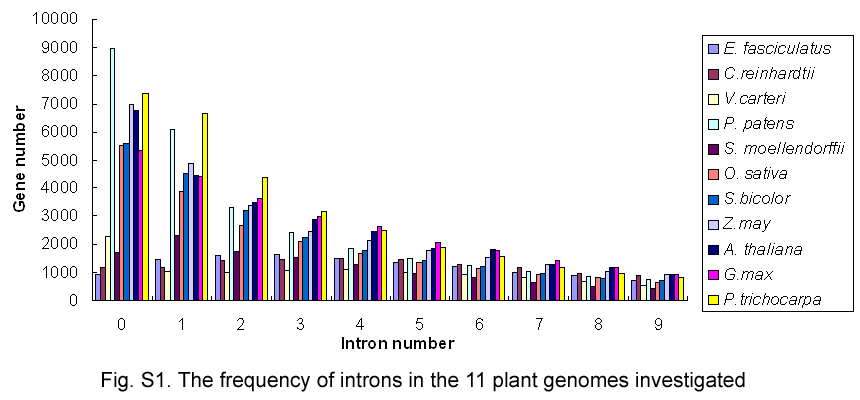

Supplement: Additional file 1: Figure S1 — The frequency of introns in the 11 plant genomes investigated. [file 1471-2164-14-56-S1.jpeg]

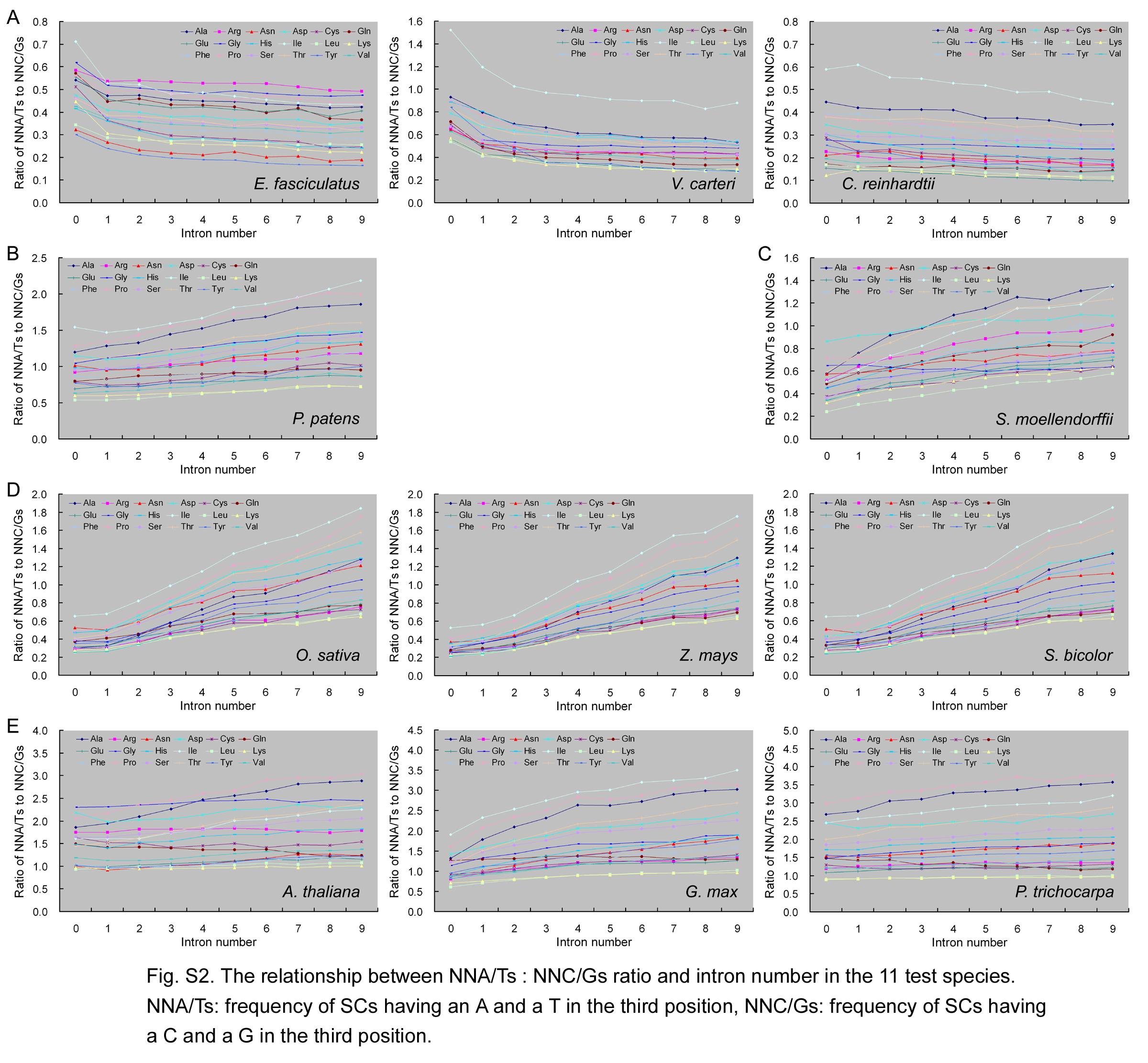

Supplement: Additional file 3: Figure S2 — The relationship between NNA/Ts : NNC/Gs ratio and intron number in the 11 test species. NNA/Ts: frequency of SCs having an A and a T in the third position, NNC/Gs: frequency of SCs having a C and a G in the third position. [file 1471-2164-14-56-S3.jpeg]

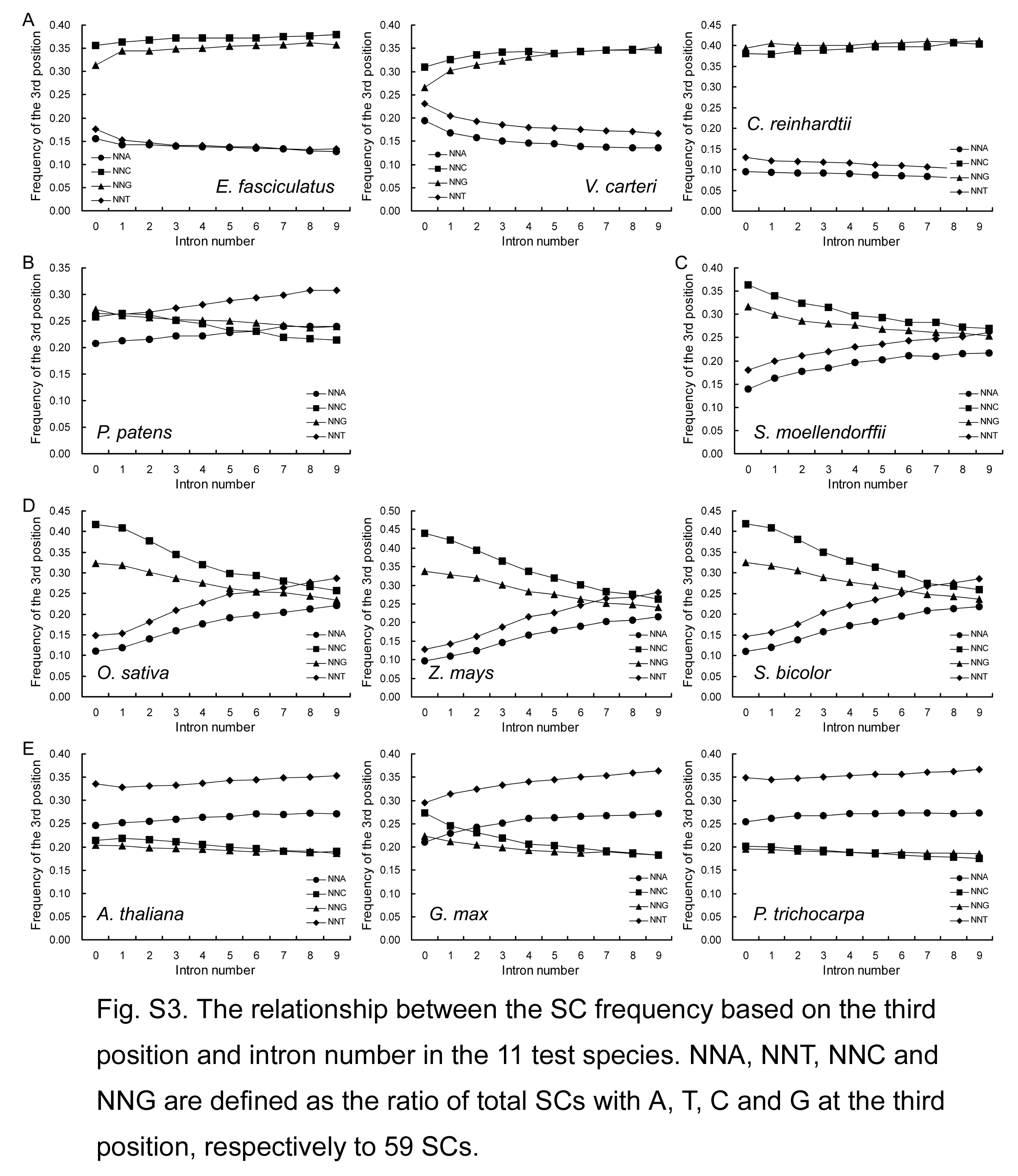

Supplement: Additional file 4: Figure S3 — The relationship between the SC frequency based on the third position and intron number in the 11 test species. NNA, NNT, NNC and NNG are defined as the ratio of total SCs with A, T, C and G at the third position, respectively to 59 SCs. [file 1471-2164-14-56-S4.jpeg]

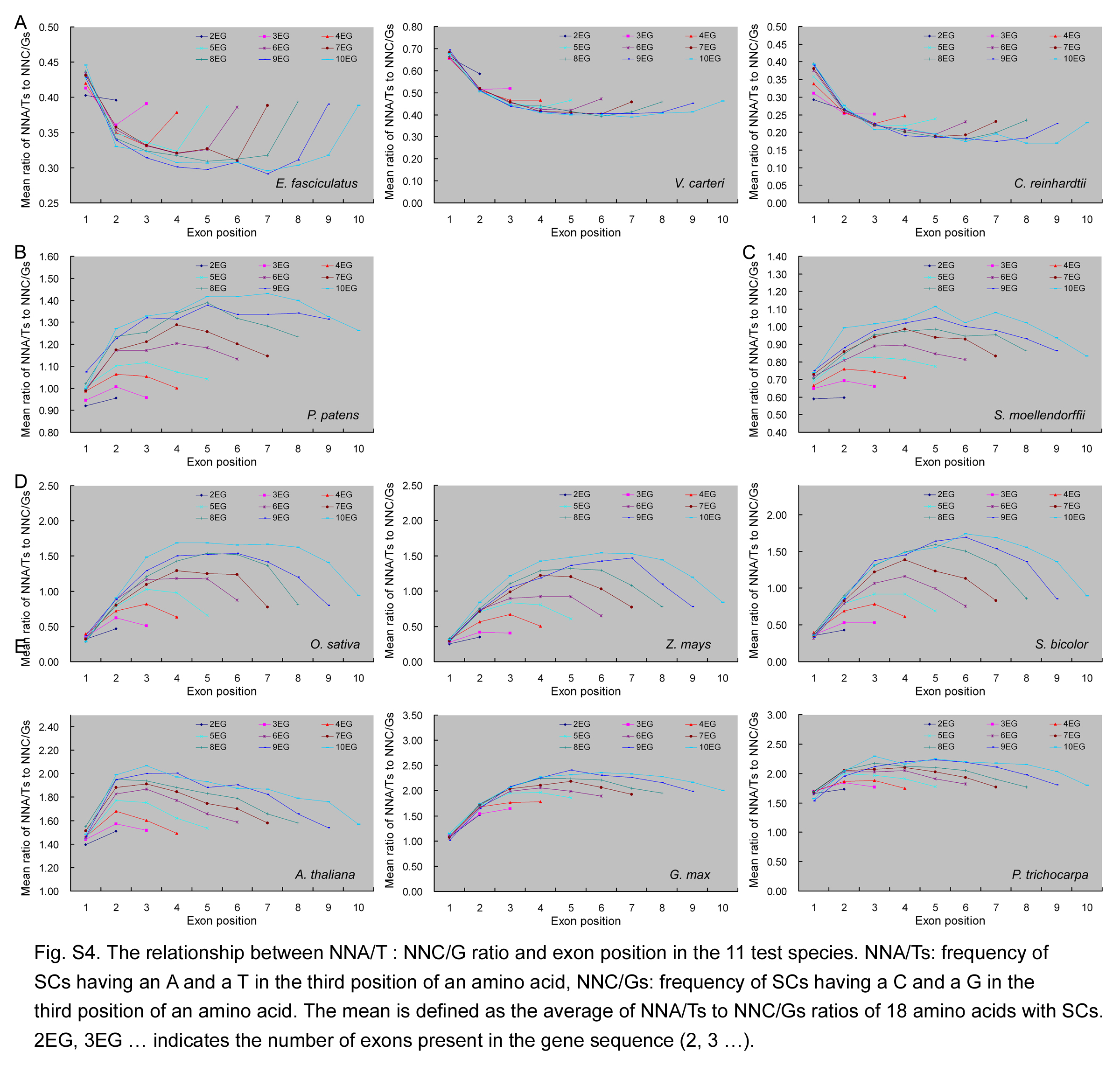

Supplement: Additional file 5: Figure S4 — The relationship between NNA/T : NNC/G ratio and exon position in the 11 test species. NNA/Ts: frequency of SCs having an A and a T in the third position of an amino acid, NNC/Gs: frequency of SCs having a C and a G in the third position of an amino acid. The mean is defined as the average of NNA/Ts to NNC/Gs ratios of 18 amino acids with SCs. 2EG, 3EG … indicates the number of exons present in the gene sequence (2, 3 …). [file 1471-2164-14-56-S5.jpeg]

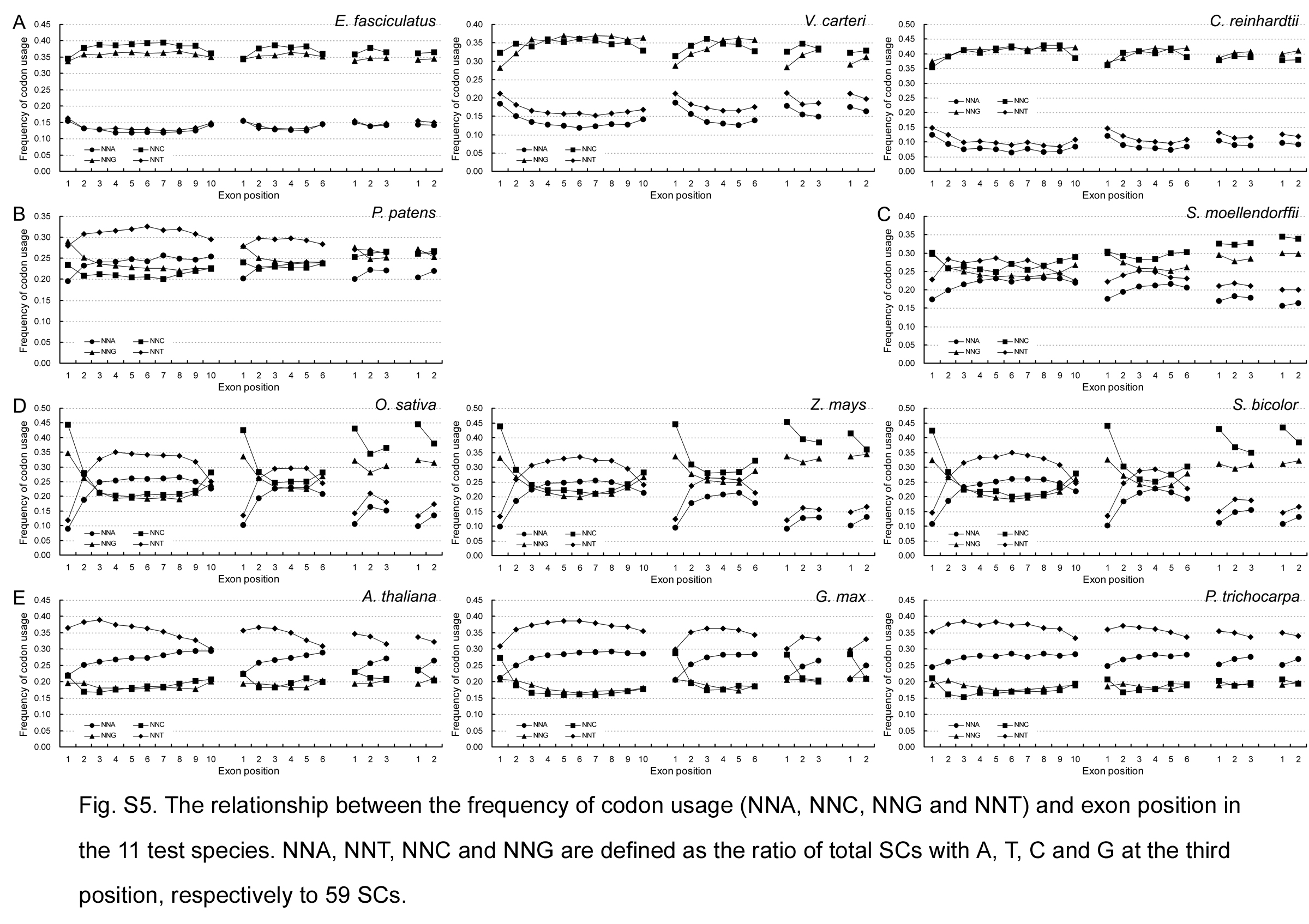

Supplement: Additional file 6: Figure S5 — The relationship between the frequency of codon usage (NNA, NNC, NNG and NNT) and exon position in the 11 test species. NNA, NNT, NNC and NNG are defined as the ratio of total SCs with A, T, C and G at the third position, respectively to 59 SCs. [file 1471-2164-14-56-S6.jpeg]

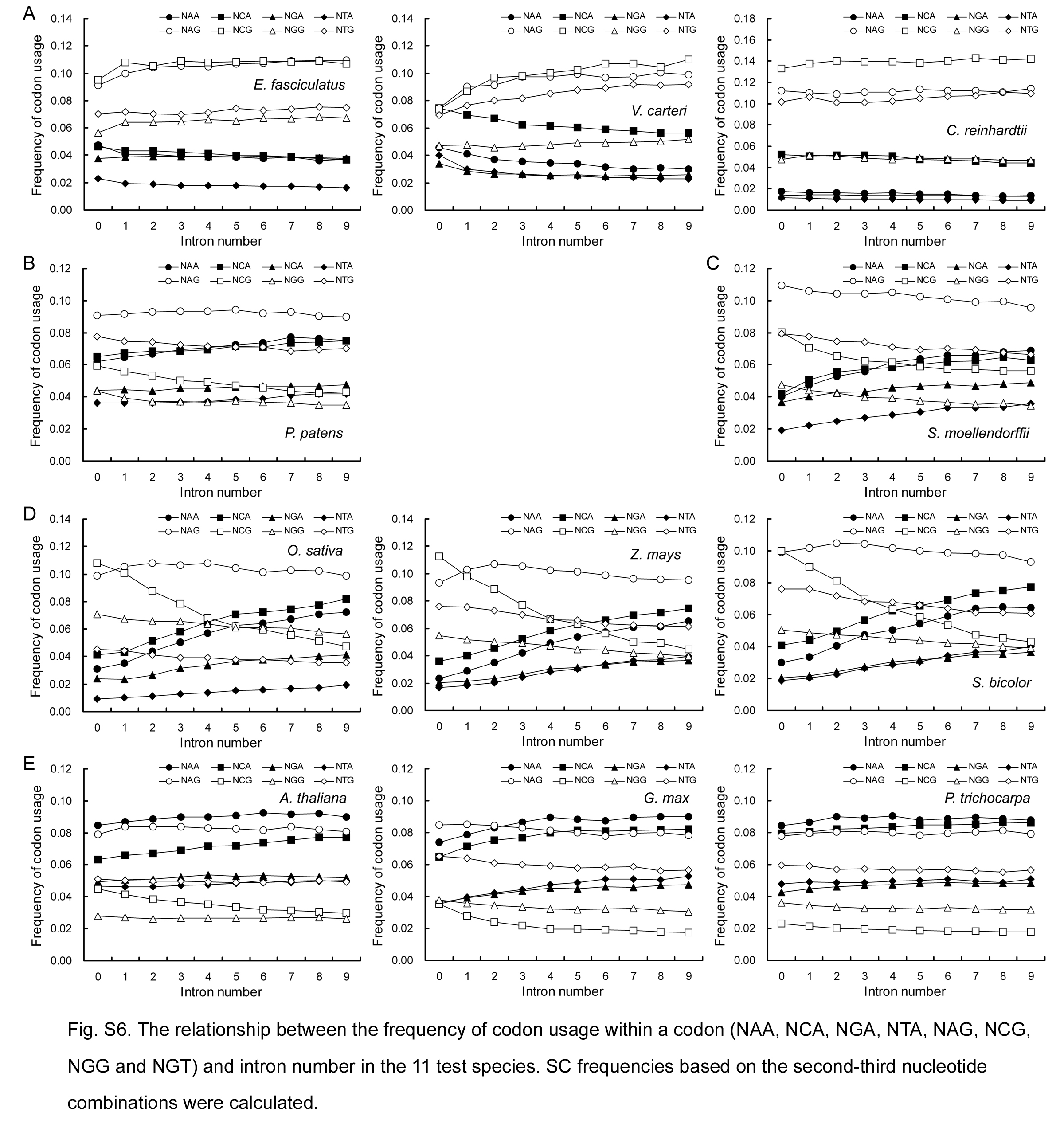

Supplement: Additional file 7: Figure S6 — The relationship between the frequency of codon usage within a codon (NAA, NCA, NGA, NTA, NAG, NCG, NGG and NGT) and intron number in the 11 test species. SC frequencies based on the second-third nucleotide combinations were calculated. [file 1471-2164-14-56-S7.jpeg]

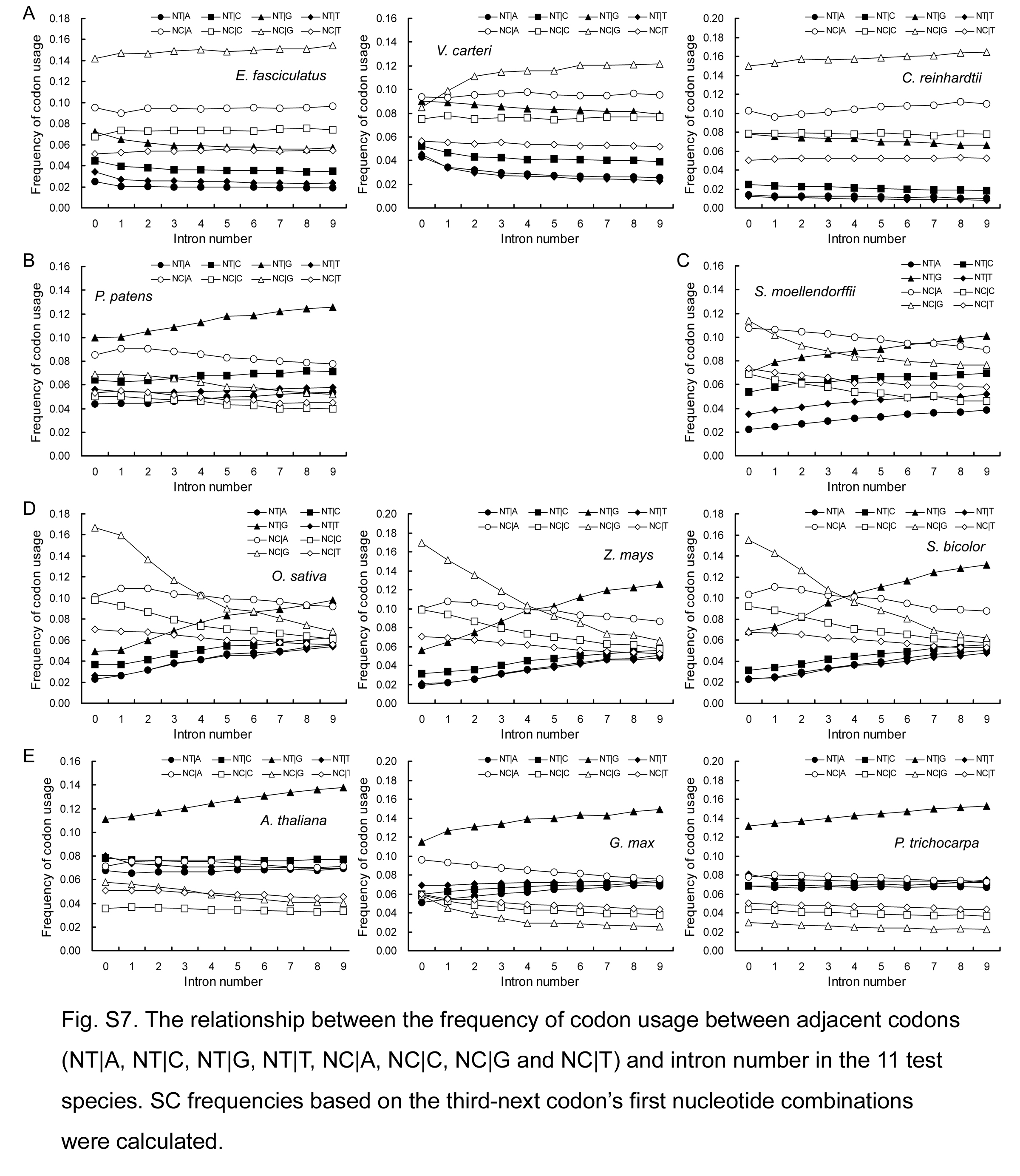

Supplement: Additional file 8: Figure S7 — The relationship between the frequency of codon usage between adjacent codons (NT|A, NT|C, NT|G, NT|T, NC|A, NC|C, NC|G and NC|T) and intron number in the 11 test species. SC frequencies based on the third-next codon’s first nucleotide combinations were calculated. [file 1471-2164-14-56-S8.jpeg]

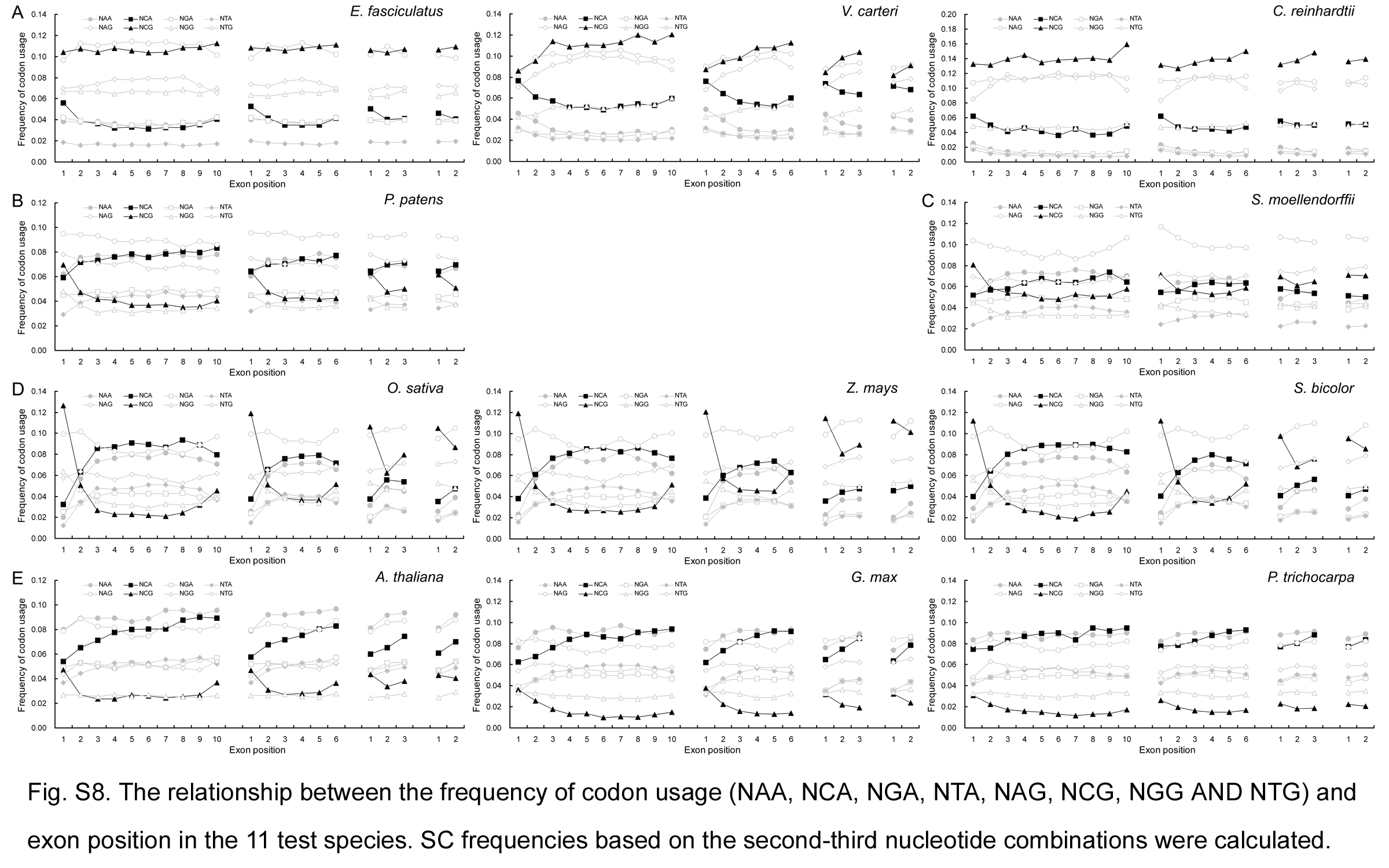

Supplement: Additional file 9: Figure S8 — The relationship between the frequency of codon usage (NAA, NCA, NGA, NTA, NAG, NCG, NGG AND NTG) and exon position in the 11 test species. SC frequencies based on the second-third nucleotide combinations were calculated. [file 1471-2164-14-56-S9.jpeg]

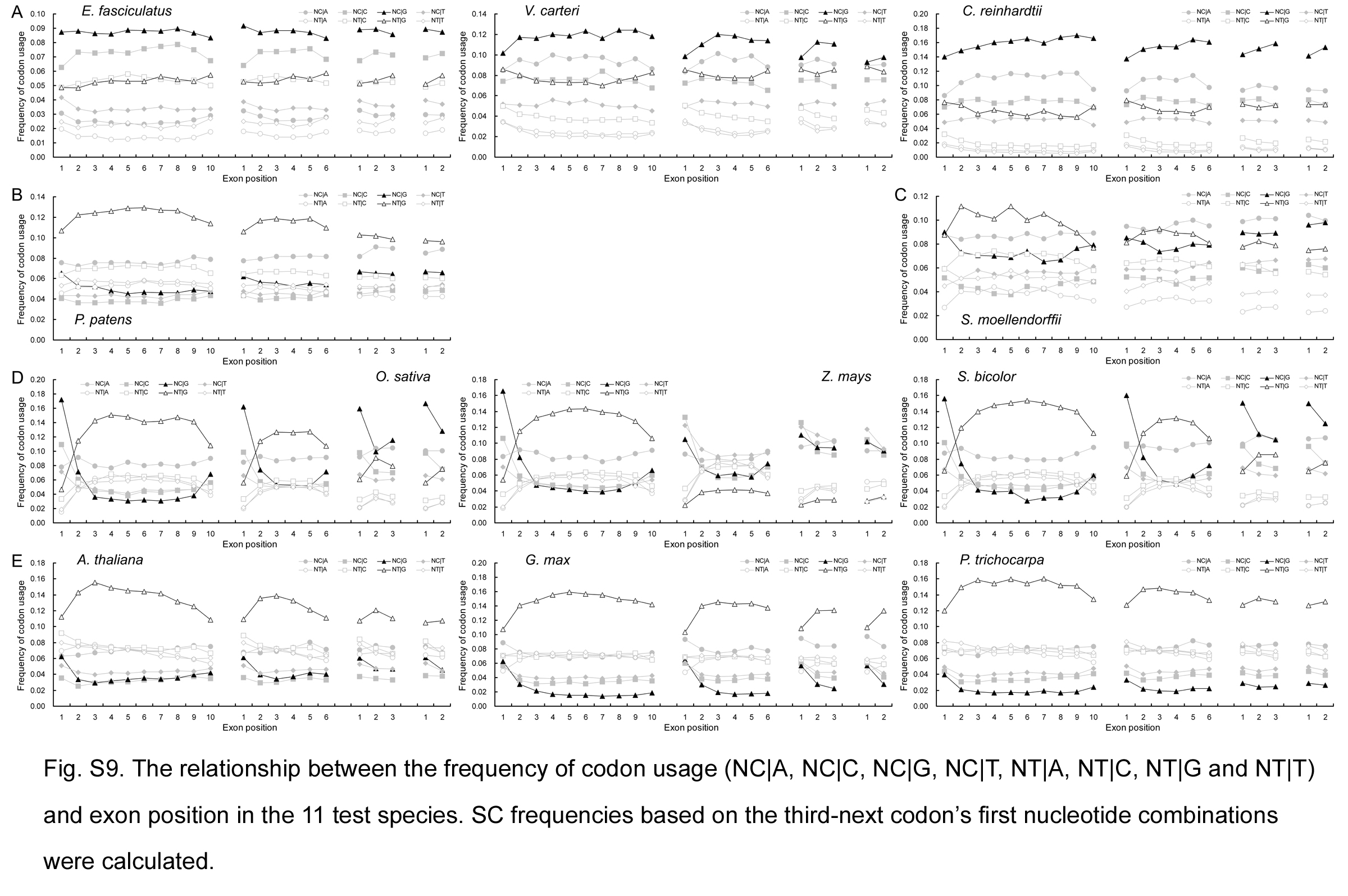

Supplement: Additional file 10: Figure S9 — The relationship between the frequency of codon usage (NC|A, NC|C, NC|G, NC|T, NT|A, NT|C, NT|G and NT|T) and exon position in the 11 test species. SC frequencies based on the third-next codon’s first nucleotide combinations were calculated. [file 1471-2164-14-56-S10.jpeg]

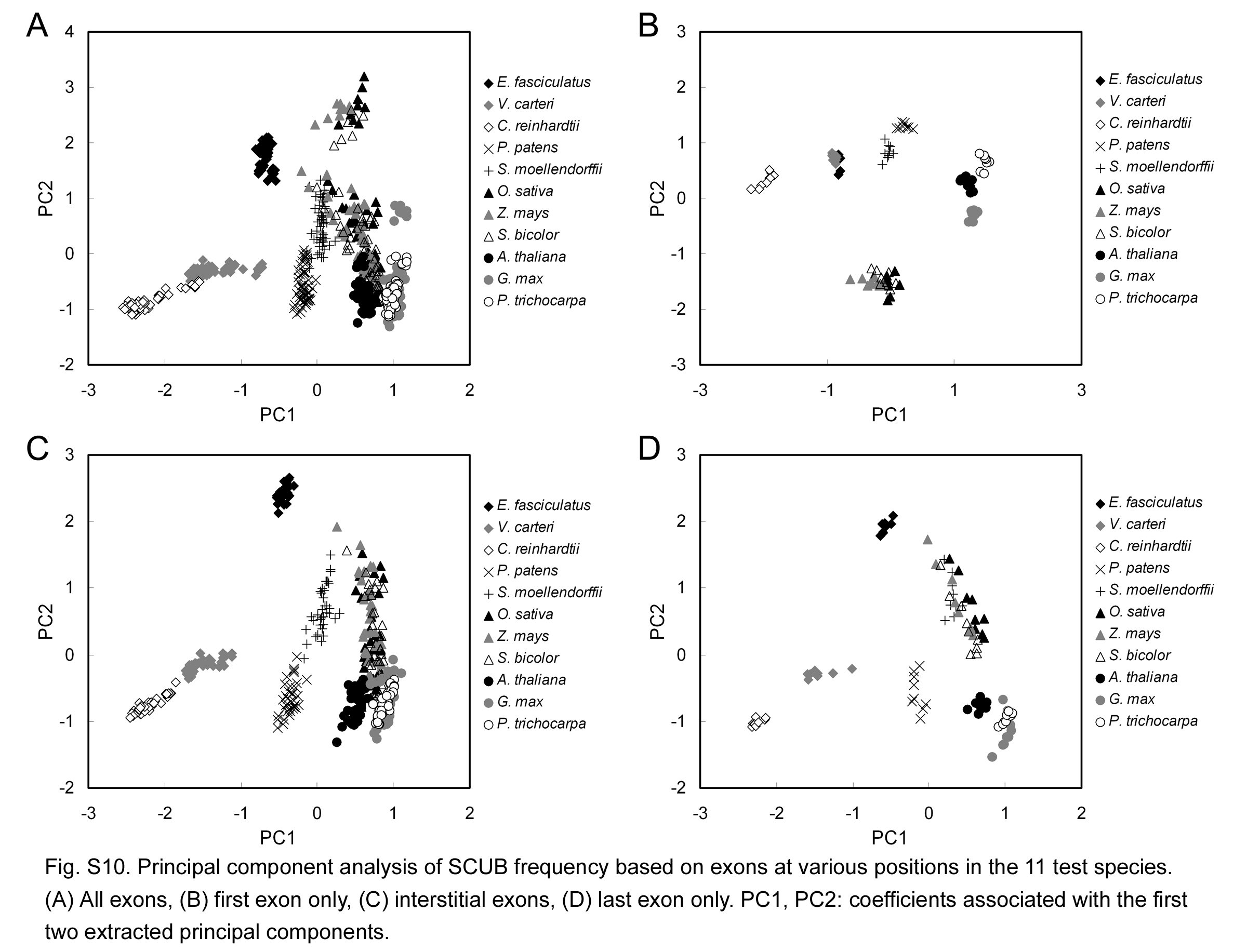

Supplement: Additional file 11: Figure S10 — Principal component analysis of SCUB frequency based on exons at various positions in the 11 test species. (A) All exons, (B) first exon only, (C) interstitial exons, (D) last exon only. PC1, PC2: coefficients associated with the first two extracted principal components. [file 1471-2164-14-56-S11.jpeg]
