# Supplementary material for: Synonymous codon usage bias is correlative to intron number and shows disequilibrium among exons in plants
Source: BMC Genomics. 2013 Jan 28;14:56. doi: 10.1186/1471-2164-14-56 (PMC3576282; doi:10.1186/1471-2164-14-56)
Supplement: Additional file 2: Table S1 — The correlation between SCUB frequency and intron number. [file 1471-2164-14-56-S2.pdf]

Table S1. The correlation analysis of synonymous codon usage frequency to intron number

amino acid	codon	*E. fasciculatus*		*V.carteri*		*C.reinhardtii*		*P. patens*		*S. moellendorffii*		*O. sativa*		*Z. mays*		*S. bicolor*		*A. thaliana*		*G. max*		*P.trichocarpa*	
		r	s	*r*	s	*r*	s	*r*	s	*r*	s	*r*	s	*r*	s	*r*	s	*r*	s	*r*	s	*r*	s
Ala	GCA	-0.913	-0.002	-0.886	-0.008	-0.980	-0.003	0.979	0.005	0.891	0.009	0.983	0.018	0.991	0.018	0.989	0.018	0.978	0.008	0.871	0.009	0.944	0.003
Ala	GCC	0.888	0.001	0.825	0.004	0.907	0.002	-0.966	-0.004	-0.965	-0.010	-0.986	-0.018	-0.992	-0.018	-0.992	-0.018	-0.939	-0.004	-0.909	-0.009	-0.986	-0.004
Ala	GCG	0.749	0.003	0.949	0.009	0.905	0.003	-0.968	-0.008	-0.890	-0.011	-0.982	-0.020	-0.989	-0.021	-0.989	-0.020	-0.950	-0.007	-0.873	-0.008	-0.856	-0.002
Ala	GCT	-0.815	-0.002	-0.946	-0.004	-0.953	-0.003	0.978	0.008	0.955	0.011	0.984	0.021	0.991	0.021	0.992	0.020	0.902	0.003	0.910	0.009	0.926	0.002
Arg	AGA	-0.485	-0.001	-0.830	-0.005	-0.941	-0.001	0.720	0.001	0.903	0.008	0.972	0.016	0.984	0.017	0.974	0.015	0.192	0.000	0.760	0.005	-0.069	0.000
Arg	AGG	0.986	0.002	-0.860	-0.003	-0.821	-0.001	-0.882	-0.002	-0.936	-0.003	0.706	0.004	0.789	0.007	0.781	0.005	-0.056	0.000	-0.968	-0.006	-0.986	-0.004
Arg	CGA	0.373	0.001	-0.647	-0.001	-0.539	0.000	0.952	0.002	0.911	0.003	0.966	0.004	0.974	0.004	0.990	0.004	0.548	0.001	0.985	0.004	0.881	0.001
Arg	CGC	-0.863	-0.002	0.345	0.001	0.802	0.003	-0.952	-0.003	-0.915	-0.007	-0.954	-0.018	-0.960	-0.021	-0.963	-0.019	0.491	0.000	-0.846	-0.006	0.136	0.000
Arg	CGG	0.757	0.003	0.899	0.009	0.385	0.001	-0.629	-0.001	-0.871	-0.006	-0.952	-0.009	-0.983	-0.011	-0.980	-0.010	-0.184	0.000	-0.089	0.000	0.692	0.001
Arg	CGT	-0.854	-0.003	-0.682	-0.002	-0.901	-0.003	0.966	0.003	0.987	0.005	0.969	0.004	0.991	0.005	0.994	0.005	-0.542	-0.001	0.994	0.003	0.863	0.002
Asn	AAC	0.882	0.008	0.895	0.010	0.925	0.005	-0.945	-0.009	-0.966	-0.009	-0.981	-0.025	-0.985	-0.030	-0.979	-0.026	-0.935	-0.009	-0.981	-0.019	-0.978	-0.006
Asn	AAT	-0.882	-0.008	-0.895	-0.010	-0.925	-0.005	0.945	0.009	0.966	0.009	0.981	0.025	0.985	0.030	0.979	0.026	0.935	0.009	0.981	0.019	0.978	0.006
Asp	GAC	0.875	0.005	0.896	0.009	0.984	0.007	-0.959	-0.009	-0.934	-0.006	-0.971	-0.032	-0.983	-0.037	-0.983	-0.033	-0.777	-0.003	-0.962	-0.012	-0.864	-0.003
Asp	GAT	-0.875	-0.005	-0.896	-0.009	-0.984	-0.007	0.959	0.009	0.934	0.006	0.971	0.032	0.983	0.037	0.983	0.033	0.777	0.003	0.962	0.012	0.864	0.003
Cys	TGC	0.855	0.012	0.609	0.007	0.888	0.006	-0.943	-0.010	-0.971	-0.012	-0.979	-0.023	-0.985	-0.027	-0.992	-0.024	0.390	0.001	-0.970	-0.011	-0.389	-0.001
Cys	TGT	-0.855	-0.012	-0.609	-0.007	-0.888	-0.006	0.943	0.010	0.971	0.012	0.979	0.023	0.985	0.027	0.992	0.024	-0.390	-0.001	0.970	0.011	0.389	0.001
Gln	CAA	-0.880	-0.008	-0.858	-0.014	-0.878	-0.002	0.959	0.005	0.961	0.015	0.972	0.019	0.983	0.023	0.988	0.019	-0.935	-0.005	0.071	0.000	-0.961	-0.007
Gln	CAG	0.880	0.008	0.858	0.014	0.878	0.002	-0.959	-0.005	-0.961	-0.015	-0.972	-0.019	-0.983	-0.023	-0.988	-0.019	0.935	0.005	-0.071	0.000	0.961	0.007
Glu	GAA	-0.797	-0.006	-0.914	-0.013	-0.991	-0.005	0.981	0.006	0.952	0.016	0.976	0.024	0.978	0.026	0.979	0.023	0.953	0.005	0.926	0.009	0.777	0.003
Glu	GAG	0.797	0.006	0.914	0.013	0.991	0.005	-0.981	-0.006	-0.952	-0.016	-0.976	-0.024	-0.978	-0.026	-0.979	-0.023	-0.953	-0.005	-0.926	-0.009	-0.777	-0.003
Gly	GGA	-0.567	-0.001	-0.810	-0.004	-0.753	-0.001	0.950	0.004	0.949	0.010	0.985	0.015	0.988	0.016	0.989	0.015	0.822	0.003	0.918	0.009	0.887	0.006
Gly	GGC	0.116	0.000	0.679	0.007	0.950	0.003	-0.928	-0.005	-0.978	-0.009	-0.978	-0.026	-0.985	-0.027	-0.989	-0.026	-0.350	0.000	-0.934	-0.006	-0.935	-0.002
Gly	GGG	0.891	0.004	-0.228	-0.001	-0.643	-0.001	-0.817	-0.004	-0.957	-0.008	-0.944	-0.003	-0.974	-0.004	-0.973	-0.002	-0.760	-0.001	-0.929	-0.006	-0.839	-0.004
Gly	GGT	-0.791	-0.004	-0.700	-0.002	-0.879	-0.002	0.988	0.005	0.980	0.007	0.978	0.013	0.989	0.015	0.992	0.014	-0.644	-0.001	0.933	0.003	0.127	0.000
His	CAC	0.916	0.010	0.937	0.014	0.982	0.008	-0.965	-0.011	-0.928	-0.016	-0.975	-0.029	-0.986	-0.034	-0.987	-0.029	-0.957	-0.007	-0.949	-0.018	-0.962	-0.005
His	CAT	-0.916	-0.010	-0.937	-0.014	-0.982	-0.008	0.965	0.011	0.928	0.016	0.975	0.029	0.986	0.034	0.987	0.029	0.957	0.007	0.949	0.018	0.962	0.005
Ile	ATA	-0.846	-0.003	-0.809	-0.012	-0.760	-0.002	0.831	0.002	0.967	0.010	0.985	0.011	0.980	0.013	0.983	0.012	0.978	0.005	0.918	0.006	0.890	0.003
Ile	ATC	0.838	0.009	0.855	0.013	0.979	0.008	-0.973	-0.010	-0.974	-0.025	-0.985	-0.030	-0.986	-0.035	-0.991	-0.031	-0.978	-0.010	-0.929	-0.011	-0.981	-0.005
Ile	ATT	-0.821	-0.007	-0.145	0.000	-0.985	-0.006	0.967	0.008	0.973	0.015	0.980	0.019	0.989	0.022	0.992	0.019	0.945	0.005	0.923	0.005	0.951	0.002
Leu	CTA	-0.762	-0.001	-0.875	-0.002	-0.953	-0.001	0.923	0.001	0.973	0.004	0.978	0.005	0.989	0.005	0.990	0.005	0.660	0.001	0.867	0.002	0.116	0.000
Leu	CTC	0.666	0.002	-0.826	-0.001	-0.955	-0.002	-0.984	-0.005	-0.977	-0.014	-0.976	-0.023	-0.987	-0.022	-0.988	-0.020	-0.987	-0.005	-0.942	-0.011	-0.978	-0.003
Leu	CTG	0.881	0.005	0.898	0.015	0.988	0.009	-0.987	-0.004	-0.967	-0.008	-0.975	-0.008	-0.982	-0.014	-0.987	-0.012	0.989	0.003	0.804	0.001	0.912	0.002
Leu	CTT	-0.773	-0.002	-0.978	-0.006	-0.993	-0.003	0.973	0.005	0.973	0.009	0.982	0.012	0.986	0.013	0.990	0.012	0.945	0.004	0.918	0.004	0.971	0.002
Leu	TTA	-0.926	-0.001	-0.820	-0.004	-0.929	-0.001	0.976	0.002	0.989	0.004	0.988	0.006	0.992	0.007	0.994	0.006	-0.182	0.000	0.965	0.005	0.678	0.000
Leu	TTG	-0.862	-0.003	-0.676	-0.002	-0.929	-0.003	-0.532	-0.001	0.947	0.004	0.970	0.008	0.991	0.010	0.992	0.009	-0.736	-0.001	-0.935	-0.002	-0.738	-0.001

Lys	AAA	-0.830	-0.010	-0.849	-0.012	-0.766	-0.003	0.970	0.006	0.967	0.015	0.982	0.020	0.987	0.023	0.991	0.020	0.745	0.001	0.943	0.008	0.941	0.002
Lys	AAG	0.830	0.010	0.849	0.012	0.766	0.003	-0.970	-0.006	-0.967	-0.015	-0.982	-0.020	-0.987	-0.023	-0.991	-0.020	-0.745	-0.001	-0.943	-0.008	-0.941	-0.002
Phe	TTC	0.811	0.010	0.803	0.011	0.967	0.008	-0.950	-0.011	-0.984	-0.018	-0.985	-0.028	-0.992	-0.032	-0.994	-0.028	-0.904	-0.006	-0.970	-0.015	-0.944	-0.004
Phe	TTT	-0.811	-0.010	-0.803	-0.011	-0.967	-0.008	0.950	0.011	0.984	0.018	0.985	0.028	0.992	0.032	0.994	0.028	0.904	0.006	0.970	0.015	0.944	0.004
Pro	CCA	-0.903	-0.004	-0.942	-0.007	-0.939	-0.002	0.961	0.004	0.848	0.006	0.976	0.017	0.986	0.020	0.985	0.018	0.920	0.003	0.822	0.007	-0.387	0.000
Pro	CCC	0.921	0.002	0.891	0.003	0.945	0.005	-0.974	-0.006	-0.935	-0.008	-0.978	-0.008	-0.987	-0.012	-0.993	-0.010	-0.024	0.000	-0.940	-0.007	-0.905	-0.002
Pro	CCG	0.749	0.002	0.849	0.010	0.571	0.001	-0.977	-0.007	-0.901	-0.009	-0.982	-0.027	-0.985	-0.027	-0.983	-0.026	-0.977	-0.009	-0.840	-0.006	-0.934	-0.003
Pro	CCT	-0.589	-0.001	-0.904	-0.006	-0.970	-0.003	0.981	0.009	0.956	0.010	0.985	0.018	0.987	0.019	0.987	0.018	0.951	0.006	0.955	0.007	0.978	0.005
Ser	AGC	0.712	0.003	0.516	0.002	-0.611	-0.002	-0.971	-0.004	-0.975	-0.007	-0.974	-0.008	-0.976	-0.009	-0.969	-0.009	0.071	0.000	-0.978	-0.002	-0.816	-0.002
Ser	AGT	-0.850	-0.002	-0.707	-0.002	-0.624	0.000	0.981	0.004	0.931	0.004	0.973	0.008	0.990	0.009	0.978	0.007	0.963	0.002	0.958	0.004	0.946	0.001
Ser	TCA	-0.903	-0.002	-0.910	-0.003	-0.943	-0.001	0.984	0.004	0.918	0.006	0.977	0.012	0.985	0.014	0.986	0.013	0.985	0.004	0.894	0.005	0.946	0.002
Ser	TCC	0.847	0.002	0.969	0.003	0.919	0.003	-0.990	-0.004	-0.921	-0.004	-0.961	-0.011	-0.981	-0.014	-0.978	-0.012	-0.976	-0.003	-0.939	-0.008	-0.887	-0.002
Ser	TCG	0.658	0.001	0.931	0.004	0.891	0.002	-0.964	-0.004	-0.888	-0.005	-0.965	-0.012	-0.972	-0.012	-0.969	-0.011	-0.980	-0.004	-0.859	-0.004	-0.948	-0.002
Ser	TCT	-0.595	-0.001	-0.890	-0.004	-0.930	-0.001	0.972	0.004	0.979	0.007	0.971	0.011	0.985	0.013	0.981	0.011	0.692	0.001	0.931	0.005	0.954	0.002
Thr	ACA	-0.898	-0.004	-0.875	-0.009	-0.520	-0.001	0.942	0.004	0.936	0.011	0.986	0.020	0.991	0.021	0.990	0.020	0.980	0.007	0.939	0.010	0.887	0.004
Thr	ACC	0.626	0.002	0.870	0.004	0.749	0.002	-0.950	-0.005	-0.975	-0.011	-0.986	-0.017	-0.992	-0.017	-0.991	-0.016	-0.977	-0.005	-0.944	-0.013	-0.973	-0.006
Thr	ACG	0.873	0.004	0.927	0.008	0.858	0.002	-0.963	-0.006	-0.849	-0.007	-0.974	-0.020	-0.983	-0.024	-0.985	-0.021	-0.970	-0.008	-0.893	-0.005	-0.915	-0.002
Thr	ACT	-0.811	-0.002	-0.947	-0.003	-0.979	-0.003	0.979	0.008	0.935	0.007	0.970	0.018	0.983	0.019	0.986	0.018	0.976	0.006	0.917	0.008	0.969	0.004
Tyr	TAC	0.893	0.008	0.887	0.023	0.959	0.007	-0.933	-0.009	-0.983	-0.012	-0.980	-0.028	-0.992	-0.033	-0.987	-0.029	-0.940	-0.007	-0.980	-0.016	-0.819	-0.003
Tyr	TAT	-0.893	-0.008	-0.887	-0.023	-0.959	-0.007	0.933	0.009	0.983	0.012	0.980	0.028	0.992	0.033	0.987	0.029	0.940	0.007	0.980	0.016	0.819	0.003
Val	GTA	-0.949	-0.003	-0.808	-0.004	-0.943	-0.002	0.982	0.003	0.984	0.005	0.986	0.008	0.989	0.009	0.988	0.009	0.968	0.003	0.950	0.008	0.872	0.002
Val	GTC	-0.350	0.000	-0.478	-0.001	-0.966	-0.004	-0.960	-0.004	-0.972	-0.005	-0.984	-0.016	-0.991	-0.016	-0.991	-0.015	-0.869	-0.003	-0.837	-0.003	-0.925	-0.002
Val	GTG	0.907	0.006	0.972	0.012	0.979	0.008	-0.968	-0.006	-0.970	-0.012	-0.980	-0.014	-0.989	-0.017	-0.990	-0.016	-0.886	-0.003	-0.984	-0.011	-0.985	-0.004
Val	GTT	-0.843	-0.003	-0.955	-0.006	-0.958	-0.003	0.967	0.007	0.976	0.012	0.984	0.022	0.992	0.024	0.992	0.022	0.812	0.003	0.986	0.007	0.930	0.004

Notes: 1. The genes with 0-9 introns were selected for analysis.
2. 59 synonymous codons referring to 18 amino acids except for Met and Trp were used for calculation.
3. *r* : relation coefficient; s: slope of linear correlation formula.
